# Healing in a Social Context: The Importance of Clinician and Patient Relationship

**DOI:** 10.3389/fpain.2021.684768

**Published:** 2021-06-10

**Authors:** Bruce E. Wampold

**Affiliations:** ^1^Modum Bad Psychiatric Center, Research Institute, Vikersund, Norway; ^2^Department of Counseling Psychology, University of Wisconsin-Madison, Madison, WI, United States

**Keywords:** healing, placebo, clinical relationship, social healing, psychotherapy, coregulation

## Abstract

When a patient presents to a health provider, the course of the disorder is composed of three effects: natural effects, specific effects, and contextual effects. Part of the contextual effect is due to the relationship between the healer and the patient. Social healing appears to be present in eusocial species and particularly well-developed in humans. Evidence for the importance of the relationship in healing is found in placebo studies, including placebo analgesics, medicine, and psychotherapy. Although the theory for how the relationship is therapeutic is not well-developed, four possible mechanisms are discussed. The implications for health care and the treatment of pain are discussed.

In 2017, the total expenditures for health care related spending in the United States, according to the Centers for Medicare and Medicaid Services, was 3.5 trillion dollars, which accounted for almost 18 percent of the United States gross domestic product. The incremental cost of health care due to pain is ~300 billion dollars (1). Advances in medicine and public health have led to a dramatic rise in longevity, with life expectancy of nearly 80 years currently compared to around 40 years a century ago. When we think of advances in medicine, we think of various medications, surgeries, and vaccinations that are important for curing or preventing diseases and improving the quality of life. Many cancers are no longer death sentences. Gastric ulcers due to H. Pylori bacteria are successfully treated with antibiotics and medication to block acid production (histamine blockers or proton pump inhibitors), supplanting relatively ineffective treatments consisting of diets of bland foods and antacid tablets.

A misconception of medicine and other healing practices is the notion that the benefits of medical interventions primarily are due to technological advances and the specific ingredients or procedures found in those advances. The benefits experienced by patients who present to a clinician for relief are not due exclusively to the specific ingredients of the treatment, as the context in which the treatment is given produces a relatively large effect as well. In this article, the focus will be on the effects of the relationship between the patient and the clinician on health outcomes, examining its relative contribution to distress relief and disease cure or management.

## Components of Healing

An individual presenting to a clinician for a health-related problem seeks relief of the distress (symptom reduction), better health (cure of disease or disease management), and/or better quality of life. It is useful to identify the various factors that are responsible for the change in health status, particularly natural effects, specific effects, and contextual effects.

### Natural Effects, Specific Effects, and Contextual Effects

There are three effects that compose humans' response to disease and injury, which here are labeled *natural effects, contextual effects*, and *specific effects*. Natural effects refer to the change in the patient's status due to the natural course of disease. Humans have biological mechanisms to protect the organism from disease and to aid in healing, including immune functions, blood coagulation, barriers such as skin, and so forth. When an individual is exposed to a pathogen or is injured, the organism often heals without intervention, a phenomenon referred to as *natural healing* (2). Of course, the natural course might involve deterioration (i.e., negative natural effect), say for Parkinson's disease.

There are effects due to the specific medications or procedures administered by the clinician. Removing the appendix, for example, will reduce symptoms and eliminate the sequelae of a bacterial infection of the appendix and will avoid a rupture. Antibiotics specifically destroy bacteria causing an illness or retard their growth. The *specific effects*, sometimes referred to as *technological healing* (2), typically characterize our views of the advances of modern medicine.

The final component of healing involves *contextual effects*. Contextual effects involve a host of factors, including patient expectations, symbolic meaning of a healing setting (e.g., a physician's white coat, syringes, diplomas on the wall), the relationship between the healer and the patient, conditioned responses to various medication or procedures, and so forth, as described by Di Blasi et al. [(3); see also (4)]. Miller et al. (2) have referred to the benefits of this component as *interpersonal healing*, but there are several aspects of this effect that involve aspects of the healing setting that do not necessarily involve an interpersonal relationship, such as the patient's response to a syringe as a healing symbol (4). Clearly, much of what is categorized as factors in the contextual effect are the factors that make placebos effective (2, 5–8), but the effect technically is not a placebo effect because in these examples, and for the most part in clinical practice, no placebo has been administered. Benedetti (9) has called such effects *placebo-like effects*.

### Effects in Context—Considerations

In 1977, Engel recognized the limitation of an exclusively biological system of medicine and proposed a model that included psychological and social factors (i.e., factors included in the contextual effect), a model that is referred to as the *biopsychosocial model* of medicine. At the turn of the century, based on the efforts of Sackett, the Institute of Medicine defined *evidence-based medicine* as “the integration of best research evidence with clinical expertise and patient values” [(10), p. 142; see also (11)], recognizing that clinical practice based solely on biology was not sufficient in terms of quality of care. Despite these efforts, the psychological and social aspects of medicine are largely ignored in the literature. Compared to the hundreds of thousands of clinical trials examining various biological based treatments, recently Di Blasi et al. (3) found 25 trials that investigated “context effects” and Kelley et al. (12) found 13 trials that estimated the effect of relationship on health outcomes.

The focus on the specific effects has been central to medicine since the origin of modern Western medicine (13). In the mid-twentieth century, the randomized placebo control group was developed to estimate the effects due to the specific factors of treatment, over and above what was produced by psychological and social factors (i.e., the placebo) and controlling for natural effects (13, 14). Indeed, the Food and Drug Administration requires superiority of a medication to a placebo for drug approval (15). When it is reasonable to expect that contextual effects exist, they account for a sizable proportion of the treatment effect and can be in some cases larger than the specific effect (16, 17).

A final issue that needs clarifying is how the effects are produced conjointly. Optimal healing is a complex combination of natural effects, contextual effects, and specific effects. Consider acute pain resulting from a surgical intervention. The incision will be sutured (specific effect) and then the tissues will progress through stages as they heal naturally, analgesics will be administered to reduce the pain until the natural course of healing has progressed sufficiently. The effect of the analgesics has both a specific effect and a contextual effect. Amanzio et al. (18) demonstrated that post-operative patients experienced significantly less pain relief when they were unaware that they were receiving strong opioid analgesics automatically dispensed with a programmed infusion machine than when the same dose was dispensed by the machine but with a physician present who told the patient that “the medication was a powerful painkiller.” (p. 206). Clearly, post-operative pain relief is a result of natural, specific, and contextual effects.

## Social Healing

From the beginning of human civilization, there have existed a variety of healing practices, involving an interpersonal relationship between a socially sanctioned healer and a person in distress (13, 19, 20). Until the advent of modern medicine in the twentieth century, the rituals involved in these healing practices often produced null or negative specific effects [i.e., the interventions were ineffective or harmful (13)], but presumably healing practices persisted over millennia due to the perceived benefits that may have been due to the contextual effects and/or misattribution of natural effects as a specific effect of the healing practice.

Social healing practices are not limited to humans but exist in other eusocial species. Remaining in close contact with infected conspecifics often creates epidemics as pathogens are communicated to healthy organisms, as is evident in the COVID-19 pandemic. There are interesting social phenomena in diseased eusocial species. For example, what might seem counter-intuitive, in ant colonies, healthy ants spend time grooming ants suffering from an infection. The grooming behavior results in a limited transmission of the pathogen from the infected ant to the healthy aunt, which elicits an immune response to the particular pathogen. The healthy ant, through social activity, acquires immunity to the pathogen, a process that has been labeled “social immunization” (21). Honeybees utilize a “social fever” when an infection is present in the colony, induced by the bees fanning their wings, which raises the temperature of the hive (22). Relevant to the current pandemic, infections can change social behavior in socially isolating ways. In highly social vampire bats, immune challenged individuals experience lethargy and fatigue, which results in decreased social contact, particularly with non-kin conspecifics (23, 24). In social species, natural healing mechanisms at the organism level have social healing analogs, which evolved to promote group fitness: “At the interface between social and individual immunity, several findings indicate that a strong social defense may replace to a certain extent the need for a sophisticated individual immune system” [(22), p. 138]. Of course, as is the case of evolved characteristics of any species, what is adaptive in the typical situation may be catastrophic as biological and environmental conditions change.

Social healing raises the question with regard to how an organism signals that it needs others to care for it and how does the conspecific recognize the signal? The signaling/recognition issue can be understood by considering pain in humans. Pain is adaptive because it indicates situations that are harmful, initiates escape from a harmful situation, and teaches the organism to avoid similar harmful situations. However, importantly, pain can be used to elicit assistance from others (25–27). The facial features associated with pain evolved relatively early in humans and are consistent across various sources of pain, including emotion pain (27, 28). The fitness benefit of the facial expression of pain is that it signals to others to elicit social assistance (27). Steinkopf (25) has proposed a signaling theory of symptoms that proposes that symptoms such as inflammation, lethargy, and pain have a signaling component: “Symptoms signal the need for care and treatment to potential helpers. Once help and treatment are granted, the signaling function is fulfilled and the symptoms diminish” (p. 1). Social support, protection, and assistance elicited by the expression of pain are beneficial to healing.

## Evidence for Social Healing: The Relationship

The healing relationship is defined as an interpersonal interaction between a clinician (or experimenter in some studies) where the clinician explains what is involved in the treatment. The relationship involves a cognitive component, where information is transmitted, as well as an emotional component that involves empathy, warmth, caring, and understanding (3, 12, 29).

The evidence for the relationship in healing is found primarily in three areas: placebos, somatic medicine, and psychotherapy. Placebo research is informative because by definition the specific effect is nil and therefore an interaction between specific factors and the relationship does not exist, making interpretation of studies less ambiguous. As well, it is relatively easy to manipulate the relationship in placebo designs. Medical/surgical investigations are informative because the importance of the relationship for health outcomes can be investigated in the setting where it is most important (i.e., when a specific treatment in administered). Finally, psychotherapy is a healing practice that involves the relationship as the vehicle by which the treatment is administered.

### Placebos

Placebos are substances or procedures without ingredients that should not, from a biological perspective, affect the health status of an individual (30). The placebos can mimic any medical intervention—there are be sham pills, inoculations, creams, and surgery. There is convincing evidence that placebos have a demonstrable effect on subjective outcomes (e.g., symptoms) as well as on the physiology of the individual, despite the lack of biological ingredients (5–8). Placebo effects have been detected in many domains, including pain (acute, chronic, as well as experimentally induced), headaches, Parkinson's disease, irritable bowel syndrome osteoarthritis, respiratory illnesses, menopausal symptoms, mental disorders (primarily anxiety and depression) among others (5–7).

In general, there is agreement that the effects of placebos “depend on a person's psychological and brain responses to the *treatment context*, which influence appraisals of future well-being” [(5); emphasis added, p. 73]. Moreover, “recent research has revealed that… psychosocial-induced biochemical changes in a patient's brain and body in turn may affect the course of a disease and the response to a therapy” [(9), p. 33]. The central characteristic of the placebo response is the psychosocial context, which includes the relationship between the patient and the clinician, the information about the intervention that is communicated to the patient, the physical healing space, the healing rituals, cultural beliefs about healing and healers, and so forth. Clearly, the discussions of placebo effects are very close, if not identical, to how contextual effects were discussed earlier. The term contextual effect is preferred generally because the effects (i.e., contextual effects) can be obtained whether or not a placebo has been administered.

The conjecture relative to placebo effects is that the relationship between the clinician and the patient augments the placebo effect obtained without the relationship. Placebo effect can be obtained by written information [i.e., providing information without a relationship, e.g., (31–33)], can occur as a conditioned response (34), and can be induced by the symbolic meaning of medical paraphernalia (4). The issue is not whether a placebo effect can be detected without a relationship but rather whether the placebo response is augmented by the relationship. There are a number of well-conducted clinical trials that have examined relationship effects and placebos, as discussed elsewhere (35) and augmented and reviewed here.

The effect of the relationship was investigated in a study of placebos in the treatment of irritable bowel syndrome (IBS), conducted by Kaptchuk et al. (36). IBS, often treated in primary care, is a prevalent disorder with no known cause but a disorder that attenuates patient's quality of life. IBS has been found to be responsive to placebos and in this study the placebo used was an acupuncture placebo. The acupuncture placebo is given by a device that the patient believes pierces the skin but does not and therefore is not true acupuncture. In this study, IBS patients were randomly assigned to one of three arms: (a) a treatment-as-usual group with no acupuncture (usual treatment from a physician), (b) the sham acupuncture twice a week for 3 weeks with *limited interaction* with the acupuncturist, and (c) sham acupuncture with the same frequency but with an *augmented interaction*. The acupuncturists participated in both conditions (i.e., in a crossed design). In the limited interaction, the acupuncturist matter-of-factly explained the procedure and indicated that they had reviewed the chart, but they did not exhibit warmth or caring. The augmented interaction included a preliminary interaction, which lasted about 45 min prior to the first acupuncture procedure, and included questions about the patient's IBS symptoms, curiosity about the effects of IBS on functioning, and inquiries about how the patient understood the cause and meaning of IBS, an interaction that the researchers called “an optimal patient-practitioner relationship” (p. 3). The acupuncturists in the augmented condition, however, were not allowed to use any specific interventions or give advice.

The outcomes measured at the end of the 3 week IBS trial were symptom severity, adequate relief from distress, global improvement, and quality of life. The results showed that the limited sham acupuncture was superior to treatment-as-usual on all outcomes, as expected. However, the augmented interaction provided additional benefit over the limited interaction, on all outcomes. With regard to global improvement, 3 percent of the treatment-as-usual patients reported moderate or substantial improvement, whereas 20 percent reported the same improvement in the limited condition, and 37 percent in the augmented condition. Interestingly, the largest effect was on the quality-of-life outcome, indicating that the relationship effect may target aspects of general distress rather than particular symptoms, as suggested by Wampold and Imel (37). According to Kaptchuk et al. (36), “The magnitude of non-specific effects in the augmented arm is not only statistically significant but also clearly clinically significant in the management of irritable bowel syndrome” (p. 6), supporting the notion that the relationship effect on healing is clinically important.

In the Kaptchuk et al. (36) study, although the acupuncturists were trained to be interpersonally warm, interested, and caring, some acupuncturist may have had a more well-developed set of interpersonal skills [see (38)]. Accordingly, there would be variability among the practitioners in the quality of their relationship with the patients regardless of the training, based on their interpersonal abilities. A follow-up analysis showed that there were significant differences among the acupuncturists: Some acupuncturists achieved better outcomes, regardless of acupuncture condition, than others (39).

The variability in outcomes due to acupuncturist in the IBS study suggests that some clinicians are more effective than others, a question studied in a double-blind randomized trial of antidepressant medication (ADM) vs. pill placebo (40). This study analyzed data from the drug arms (ADM and pill placebo) of the NIMH Treatment for Depression Collaborative Research Program (NIMH TDCRP). According to the NIMH TDCRP, psychiatrists in this study were coached to “provide a generally supportive atmosphere” (p. 311) during clinical management, which included a 45 to 60 min initial session and then weekly sessions of 20 to 30 min thereafter (41). Thus, the conditions were seen as medication (verum or placebo) “plus minimal supportive therapy” (p. 311). The antidepressant intervention was superior to placebo (42); ADM vs. placebo accounted for about three percent of the variability in outcomes, approximately equal to the usual ADM effect. However, psychiatrists accounted for about nine percent of the variability in outcomes. In this study the more effective psychiatrists delivering placebo had better outcomes than the less effective psychiatrists delivering the placebo. Because this was a double-blind randomized trial, the difference among the psychiatrists was due to differences in clinical management.

In a study designed to determine the additive effects of relationship to both placebo and verum, Fuentes et al. (43) examined the effect of the relationship on pain intensity and pain sensitivity of patients with chronic low back pain. Patients received either active interferential current therapy (IFC, the verum) or sham IFC in conjunction with either a limited relationship or an enhanced relationship, resulting in a 2 (verum IFC v placebo IFC) by 2 (enhanced relationship v limited relationship). In the limited relationship condition, the practitioners introduced themselves and explained the purpose of the treatment whereas in the other condition “the therapeutic interaction was enhanced through verbal behaviors, including active listening (i.e., repeating the patient's words, asking for clarifications), tone of voice, non-verbal behaviors (i.e., eye contact, physical touch), and empathy” (p. 480). The practitioners left the room during the procedure in the limited relationship condition but they remained in the enhanced condition. For both the verum and for the placebo, the augmented relationship condition produced superior outcomes relative to the limited relationship condition. The authors concluded, “The context in which physical therapy interventions are offered has the potential to dramatically improve therapeutic effects” (p. 477).

As mentioned previously, there is a conjecture that the therapeutic relationship is composed of two components, cognitive and emotional (44, 45). Howe et al. (45) attempted to tease out these two aspects of the clinical relationship. In this study, the participants were given a physical examination, which included assessment of vital signs as well as an “allergy test,” as a screen for a subsequent purported medical study. The allergy test caused a reaction in all participants because the skin was pricked with histamine. The participants were informed that they were disqualified from the medical study and were given a sham placebo cream, which the participants were told would reduce their allergic reaction^1^. The histamine prick/placebo cream procedure was executed in four conditions—warmth (high vs. low) crossed with competence (high vs. low). High warmth involved having the physician use the participant's name, warm non-verbal behavior (eye contact, proximal seating, and smiling facial features), and inviting office furnishing (e.g., posters with calming images) and the low warmth condition had an absence of these features. In the high competence condition, the physician was verbally fluent (e.g., gave a cogent explanation delivered with confidence), the examination procedures were administered efficiently without mistakes, and the examination room was well-organized, whereas the low competence lacked these features. The rate of change in the reaction to histamine, which was assessed as wheal diameter, was the outcome measure. The wheal diameter decreased most quickly and the final wheal diameter was smallest in the high warmth/high competent condition, whereas the wheal diameter decreased most slowly and the final wheal diameter was largest in the low warmth/low competence condition The results of the mismatched conditions (low competence/high warmth and high competence/low warmth) were intermediate to the low/low and high/high conditions, suggesting that warmth and competence contributed to the effect of the placebo cream.

The final study reviewed examined pain tolerance threshold under two conditions (46). An actor portraying a physician administered placebo cream to healthy volunteers who participated in a cold-pressor test; tolerance and threshold were assessed before and after administration of the placebo. In one condition, the “physician” portrayed a traditional doctor/patient relationship and in the other the “physician” role emphasized “attentiveness and strong suggestion, elements…present in ritual healing” (p. 1). The latter condition, emphasizing attentiveness and suggestion, resulted in increased tolerance and threshold. The authors concluded that a “structured manipulation of physician's verbal and non-verbal performance, designed to build rapport and increase faith in treatment, is feasible and may have a significant beneficial effect on the size of the response to placebo analgesia” (p. 2).

Evidence for the importance of the clinician/patient relationship in producing a placebo effect appeared in a meta-analysis of randomized clinical trials that examined predictors of placebo analgesia response in chronic pain. In this meta-analysis placebo effects were associated with the number of face-to-face visits with the clinician; that is, studies with more face-to-face visits reported larger placebo effects (47).

The experiments that examined the relationship between the clinician and the patient when a placebo was administered found convincing evidence that a good (either warm and/or competent) relationship augments the effect of placebo. The evidence for relationship is particularly strong because placebos contain no specific ingredients that could interact with the relationship to produce better outcomes.

### Somatic Medicine and Health Service

The evidence for the effects of relationship in the medical/surgical literature is less straightforward, primarily due to the paucity of such research. In 2001, Di Blasi et al. (3) conducted a review of context effects on health outcomes, some of which examined the relationship as a contextual factor. Their search strategy yielded only 25 trials that met inclusion criteria; the trials were rated as being predominantly poor quality (of the 25, only 5 were rated as “very good” and 6 as “good”). Of the 25 trials, 19 were classified as providing “cognitive care” but most studied the effects of provision of information rather than focusing on the quality of the relationship; generally, it was found that practitioners who attempted to influence patient's beliefs about the treatment had an effect on patients' health outcomes. No studies manipulated only emotional care, but four trials examined combining cognitive care with emotional care and the results of these studies suggested that providing information (cognitive care) in a warm and accepting way produced better health outcomes than a neutral situation. Di Balsi et al. concluded, “Practitioners who attempted to form a warm and friendly relationship with their patients, and reassured them that they would soon be better, were found to be more effective than practitioners who kept their consultations impersonal, formal, or uncertain” (p. 760). Unfortunately, insufficient statistics were reported in the primary studies to meta-analytically estimate the size of the relationship effect.

The most recent review of relationship in somatic medicine was a meta-analysis of studies that examined the effect of relationship on health (12). Inclusion criteria were that (a) studies had objective or validated subjective measures, such as pain ratings, and (b) studies that systematically manipulated the patient-clinician relationship. The aggregate standardized mean difference in favor of better relationship leading to better health outcomes was 0.11, which statistically significant, although small. The authors made the following conclusion:

This systematic review and meta-analysis of RCTs suggests that the patient-clinician relationship has a small, but statistically significant effect on healthcare outcomes…. relatively few RCTs met our eligibility criteria, and… the majority of these trials were not specifically designed to test the effect of the patient-clinician relationship on healthcare outcomes. (p. 1).

The direct evidence for a relationship effect in medicine is sparse and the quality of evidence that is present is relatively poor. In the placebo literature, several well-conducted trials of the relationship have been conducted with the stated purpose of testing the relationship effect, whereas the relationship effect has not been the object of rigorous examination within the medical literature. This might be surprising given the effect of physician-patient relationship on medical patient malpractice intentions (48).

### Psychotherapy

Although the evidence for relationship effects from psychotherapy is voluminous and persuasive (37, 49), there are logical and pragmatic limitations to the evidence for the importance of the relationship. The investigation of specific effects in medicine uses a placebo control to rule out psychosocial effects (i.e., the contextual effects) so as to isolate the biological effects (i.e., the specific effects). In the study of placebos, there are by definition no specific effects and the contextual effects can be investigated by manipulating various aspects of the context (e.g., a warm relationship vs. a cold relationship). In psychotherapy, the specific effects and the contextual effects are both produced by psychosocial factors. Classically, there has been a distinction made between the *common factors* and *specific ingredients* in the psychotherapy literature but they are logically both psychosocial effects. Psychotherapy, as a healing practice, depends on the relationship between the therapist and the patient as any therapeutic actions (i.e., so called specific ingredients) cannot be delivered without a relationship. Furthermore, common factors include the acceptance and enactment of particular therapeutic actions and consequently the contextual factors of psychotherapy involve the patient's expectations that the therapeutic actions are effective, further confounding the two types of effects. The logical problems created by the artificial distinction between these two types of effects have been thoroughly discussed (37, 50, 51).

In addition to the conceptual problems related to contextual and specific effects in psychotherapy, there are pragmatic/ethical issues. In placebo studies, there is little difficulty in manipulating relationship factors, as was evident from the studies reviewed. In medicine, the emphasis is on isolating the biological specific effect and therefore the impediments to manipulating the relationship involved with the contextual effect is not objectionable, even if it is not of particular interest. In psychotherapy studies, it is not possible to deliver the treatment without a relationship (i.e., the intervention would no longer be psychotherapy) and furthermore it is not ethically allowed to have a condition with an intentionally weakened relationship, such as assigning patients to a condition where the therapist is proscribed from being empathic.

Despite the problems designing experimental studies that examine relationship effects in psychotherapy, there are hundreds of studies that have examined the association between the degree to which a relationship factor is present and psychotherapy outcome (49). Recently, Norcross and Lambert (52) summarized the results of meta-analyses of the correlation between a relationship factor and psychotherapy outcomes. These correlations, when converted to standardized mean differences (SMD), were moderately large for many relationship variables, including the therapeutic alliance (SMD = 0.57), patient-therapist collaboration (SMD = 0.40), therapist empathy (SMD = 0.58), therapist congruence/genuineness (SMD = 0.46), the real relationship (SMD = 0.80), and addressing ruptures in the alliance (SMD = 0.62).

The therapeutic alliance, which consists of agreement about the goals and tasks of therapy as well as the bond between therapist and patient, is the most extensively studied relationship variable in psychotherapy (37, 52, 53). There are over 300 studies that have examined the alliance-outcome association, involving over 30,000 patients (53); consistently, the alliance measured early in therapy is a predictor of the outcome of therapy. There is convincing evidence that this association is not confounded by early symptom change or other factors and is important for all types of therapies (37, 53–55). Moreover, it is the therapist contribution to the alliance that is important for producing therapeutic outcomes—that is, therapists who are better able to form an alliance across a variety of patients have better outcomes than therapists whose ability to form an alliance with patients is poorer (37, 56–59).

Increasingly, various psychological treatments are being effectively delivered electronically, with minimal contact with a therapist (60). The issue for the study of relationship is not whether such treatments are effective, but whether some form of relationship, perceived by the consumer of such intervention, contributes to outcomes. Somewhat surprising is evidence that consumer rated alliance with a therapist in internet delivered treatment is associated with outcome to the same extent as it is in face-to-face psychotherapy (53, 61).

Further evidence for the importance of relationship for psychotherapy outcomes comes from the therapist effects literature. Therapist effects refers to the situation that some therapists are more effective (i.e., produce better outcomes) than other therapists, regardless of characteristics of the patients or other factors. Therapist effects in psychotherapy have been detected in randomized clinical trials as well as naturalistic setting (59, 62). Indeed, therapist effects exist within various treatments and the size of the therapist effect is greater than the between treatment effect; that is to say, the particular therapist delivering the treatment is more important than the particular treatment being delivered (37, 59). As discussed earlier, providers effects have been detected in the delivery of placebos (39) and in psychopharmacology (40).

Of interest to the present topic is the question of what are the characteristics and actions of effective psychotherapists. Research has shown that the age, gender, experience, ethnicity, profession of therapist, size of therapist caseload, self-reported social skills, interviewer's rating of trainees' clinical skill, and therapist theoretical orientation do not differentiate more effective therapists from less effective therapists (59). There is some evidence that therapist attitudes, activities outside of therapy, and burnout explain some of the difference among therapists (59). However, the most important predictors of therapist effectiveness are the interpersonal skill of the therapists displayed in interpersonally challenging situations (38, 59, 63). Anderson et al. (38) had therapists respond to video-presented challenging patient vignettes and found that *facilitative interpersonal skills* (FIS) displayed by the therapist in response to the vignette predicted the outcomes obtained by the therapists—this was the first time therapists skills assessed outside of therapy predicted therapy outcomes. The FIS include verbal fluency; therapist communication of hope and positive expectations; persuasiveness; emotional expression; warmth, acceptance, and understanding; empathy; alliance bond capacity; and alliance rupture-repair responsiveness. Anderson and colleagues' studies, as well as others who have measured similar skills in challenging situations (63), have shown that psychotherapy trainees who are better able to exhibit these skills at the beginning of training have better outcomes 2 to 5 years in the future (63, 64).

The evidence from the psychotherapy literature clearly indicates that the relationship component of the treatments is critical to successful outcomes, regardless of the treatment being delivered. That psychotherapy is as effective as medications for many mental disorders (65–67), and that the relationship is key to successful psychotherapy, provides further evidence for social healing.

## How is the Relationship Healing?—Theoretical Considerations

Although the evidence for social healing appears to be strong, the studies reviewed have not investigated the psychological mechanisms involved in producing outcomes. What is it about the relationship with a warm and competent healer that leads to better outcomes? There have been a few theoretical discussions (29, 35, 68), which are summarized here.

### Interactive Effects—Improving Adherence

One possible mechanism for the therapeutic value of relationship, which was alluded to earlier, is that the specific ingredients and aspects of the relationship interact. The most obvious way that this may happen in medicine is that a good relationship with the clinician augments patient adherence to the specific ingredients of the treatment. That is, if a patient has a good relationship with the practitioner, then the patient will follow the prescribed course of treatment, say, by taking the medication as prescribed. There is meta-analytic evidence that physician communication is positively correlated with patient adherence; there is almost a 20 percent greater risk of non-adherence if the physician communicates poorly (69, 70).

However, there is some evidence that makes interpretation of medical adherence studies ambiguous (71). Not surprisingly, patients have better outcomes if they adhere to effective drug therapies. A meta-analysis of adherence to effective drug therapy and mortality found that the odds of mortality were lower when patients used their medications as directed, not surprisingly, but interestingly odds of mortality were also lower when patients adhered to a placebo as well, suggesting the benefits of adherence might involve a contextual effect as well as a specific effect (72). Indeed, there several large clinical trials that show that adherence to placebos reduces morbidity and mortality (73, 74).

Interestingly, the interaction between the relationship and treatment has been a much-debated topic, in a slightly different guise. As discussed earlier, the alliance, measured early in psychotherapy, is a robust predictor of psychotherapy outcome. What is not clear is how the alliance is therapeutic. There is one camp who argue, with some supporting evidence, that the alliance is therapeutic by itself [i.e., independently of other therapeutic actions; see e.g., (75, 76)]. This view, which is espoused most persuasively by relational psychodynamic theorists and researchers, propose that a strong alliance, and particularly one that is “ruptured and repaired,” provides the patient a learning experience in relationships generally that then leads to better mental health. On the other hand, there are those who conceptualize the alliance as a collaborative relationship that is necessary to do the difficult work of therapy (77), a perspective that was expressed by Bordin (78), when he described the alliance as a pan-theoretical construct. This perspective is articulated most clearly by those with a cognitive-behavioral therapy (CBT) orientation, who point to evidence that agreement about the goals and tasks of therapy is predictive of outcome in CBT (79, 80). The latter perspective is an interactive effect of relationship and specific ingredients.

### Relationship Combats Loneliness

Humans, as a social species, rely on the assistance of others for survival [e.g., see (81)] Socially isolated individuals lack the social connections necessary to thrive and to survive, particularly when under threat. It is well-established that obesity, smoking, lack of exercise, excessive drinking, and failure to receive influenza vaccination, have deleterious effects on health and increase mortality. However, loneliness is a greater risk for mortality than any of these factors (82, 83). A warm, caring, and understanding clinician might well provide needed social support for patients who are socially isolated.

There are many related social isolation constructs, but the most predictive of mortality is perceived loneliness (83). Individuals may have adequate social support, but still may not feel supported by those in their network during difficult times; a caring and understanding clinician may be particularly valuable in such cases. Colbie Holderness, the first wife of former Trump White House staff secretary Rob Porter and victim of his physical abuse, poignantly made this point:

Then there is the just-as-serious issue of being believed and supported by who you choose to tell. Sometimes people don't believe you. Sometimes they have difficulty truly understanding what you are trying to tell them. Both Willoughby [Porter's second wife] and I raised our cases with clergy. Both of us had a hard time getting them to fully address the abuse taking place. It wasn't until I spoke to a professional counselor that I was met with understanding. (https://www.washingtonpost.com/opinions/rob-porter-is-my-ex-husband-heres-what-you-should-know-about-abuse/2018/02/12/3c7edcb8-1033-11e8-9065-e55346f6de81_story.html).

The importance of provider warmth, caring, and understanding during times of distress is bolstered by the evidence that placebos are most effective when distress is high and individuals are seeking relief (8).

As discussed earlier several placebo studies that varied the emotional components (warmth, caring, and empathy) of practitioners found that these characteristics augmented response to placebos. Interestingly, the study of IBS found that the largest effect was for quality of life (36), which is not symptom specific. Wampold and Imel (37) hypothesized that emotional relationship variables would affect quality-of-life and well-being domains to a greater extent than symptom measures. As well, in psychotherapy, the bond between the therapist and the patient is most predictive of the outcome of the treatment when the patient has low social support (84), which supports the conjecture that the relationship reduces feelings of loneliness and leads to better outcomes.

### Relationship Is Important for Creating Expectancies

Expectancies are thought to be central to the response to placebos. There are many ways to acquire expectancies. As discussed earlier, placebo effects, most likely due to expectancies, can be created without face-to-face interactions (32, 33), say by written information. As well, response to placebos can be conditioned or created by vicarious learning (34, 85). However, it may well be that the most efficient way that expectancies are created is through verbal persuasion.

Typically, people have an expectation that inserting a metal object, say a fork, into an electrical socket will create a painful shock. It is doubtful this was learned by classical conditioning (insertion of the knife followed by a shock, a pairing that generalized to other metal objects) or by vicarious learning (say, by observing an older sibling being shocked), although in various workshops conducted by the author, typically there are one or two participants who report that they learned to avoid inserting metal object in electrical sockets by classical conditioning or vicarious learning. Most people have learned to avoid inserting a metal object into an electrical socket in the way we learn numerous important things—someone we trust informed us about the subject. That is, the expectation of an outcome (here a negative outcome) was created by a verbal transaction with a trusted person, which is a very powerful way to generate expectancies (86).

There is support for the importance of verbally transmitted information from trusted others in various fields. Many thoughts, behaviors, feelings, preferences, and mental states spread through social networks (87); that is, individuals are influenced by those with whom they are close. Lieberman (81), in his discussion of the neuroscience of social relations, makes this clear: “Our brains are designed to be influenced by others” (p. 8). Patients are neurologically predisposed to believe in the explanations provided by a clinician, particularly if the clinician is perceived to be competent and caring. Placebo research has begun to elucidate the components of persuasive explanations on response to placebos (31). A useful framework for understanding this process is persuasion theory (88), which has been used to explain response to placebos (86).

### Relationship Result in Regulation of Emotion

Many mental health disorders are characterized by emotional dysregulation. In addition, medical patients often present with emotional distress due to worry about their medical condition as well the disruption of their lives that can result. Physiological equilibrium is needed for psychological, physical, and social well-being so attempts are made to help the patient regulate their emotions, which puts the locus of regulation on the patient. However, the physical presence of someone with whom we are close can reduce arousal and distress, a phenomenon called coregulation, social regulation, or interpersonal emotion regulation (89–91). Coregulation “refers to the process by which relationship partners form a dyadic emotional system involving an oscillating pattern of affective arousal and dampening that dynamically maintains an optimal emotional state” [(89), p. 202]. Thus, emotional regulation is conceptualized as a dyadic phenomenon rather than an individual one. There is evidence for co-regulation mechanisms. In a study of maritally satisfied women in a stressful situation, holding the hand of their husbands attenuated arousal in comparison to holding the hand of a stranger or not holding anyone's hand [(92); see also (93, 94)]. Coregulation has been detected in moment-to-moment emotional states of psychotherapists and patients (95, 96). Coregulation has been discussed as a mechanism involved in the beneficial aspects of empathy in medicine (29, 68).

## Discussion

The purpose of the present review was to present evidence that the relationship between a clinician and patient creates a sizable effect in response to treatment, which is important clinically and theoretically. Although the evidence for the effects due to the relationship is rather thin relative to evidence for the specific effects of various healing practices, further consideration of relationship effects in healing is warranted.

Although the majority of studies that have examined the relationship as a factor in healing have not involved pain as the health condition, there is good reason to believe that the relationship would be important for the treatment of pain. Comprehensive evidence exists that shows that pain is responsive to placebo interventions and that the expectations for pain relief are critical mechanisms of response to placebos [see (7), Chapter 10]. In this article, it is clear that the relationship with the healer is important for creating expectations for relief and augmenting the effect of placebos as well as specific interventions, including pain reduction or pain tolerance interventions. As well, as was mentioned earlier, awareness of receipt of analgesics through an interaction with a physician decreases the pain and increases pain tolerance (7, 18, 97).

Most obviously, more research is needed, particularly in the medical context. The focus on biological effects (i.e., separation of a pharmaceutical or procedure from a placebo) has diverted attention from what many medical providers recognize and act upon—relationship is important. In an age of cost containment and cost effectiveness, the importance of the relationship (and time to properly develop a therapeutic relationship) often is ignored. Additional research evidence would act to counter the focus on evidence-based treatments, in medicine and in mental health care, and an increased attention to harnessing the power of the relationship.

Clearly, the training of relationship skills in provider education should be emphasized. Recognition of the importance of the relationship is not sufficient and relational skills training is needed to develop expertise, in the same way that expertise is acquired in other domains (98). Moreover, medical and psychological education should consider interpersonal skill as an admission criterion. Anderson and colleagues, as well as others, have shown that the interpersonal skill of clinical psychologists when they begin their education is predictive of therapy outcomes up to 5 years in the future (63, 64).

In this review, the impression might be given that a “good” relationship is universal. Clearly, this is not the case and there are cultural and personal variations in what makes an effective relationship in a health care setting. Eye contact may be facilitative for many but for some cultural groups it is counterproductive. For some personalities and disorders, the intensity of a close relationship with a healer can be threathening and produce distress. Interpersonal relationship are complex and simple and univeral rules, such as making eye contact with a patient or calling the patient by their name, simplifies the endeavor in ways that may be ineffective and even discriminatory.

The theory underlying the relationship effects in healing is relatively underdeveloped, but clearly the healing mechanisms are psychosocial. Further research in social psychology, clinical psychology, placebo studies, medical anthropology, and pain would elucidate the mechanisms of social healing.

## Author Contributions

BW is the sole author and is responsible for all aspects of the article.

## Conflict of Interest

The author declares that the research was conducted in the absence of any commercial or financial relationships that could be construed as a potential conflict of interest.

## Footnotes

^1^There was also a condition where the participants were told that the cream would aggravate their reaction, but that part of the study is not relevant to the current discussion of placebo as opposed to nocebo.
